# YAP: The nexus between metabolism and cardiac remodeling

**DOI:** 10.1172/JCI157664

**Published:** 2022-03-15

**Authors:** Chen Gao, Yibin Wang

**Affiliations:** 1 Department of Pharmacology and Systems Physiology, University of Cincinnati, Cincinnati, Ohio, USA.; 2 Program in Cardiovascular and Metabolic Diseases, Duke-NUS, Singapore.

## Abstract

Cardiomyocyte hypertrophy is an integral part of cardiac remodeling that occurs under physiological or pathological stresses. It can lead to heart failure in a pathological form or oppose functional deterioration in a compensatory one. The mechanisms underlying an adaptive outcome of hypertrophy are ill defined. In this issue of the *JCI*, Kashihara et al. explored the role of the Yes-associated protein 1 (YAP) transcription factor in the heart, using cell culturing and mouse models. YAP activity was found to be associated with changes in genes of the glycolytic and auxiliary pathways under stress. Notably, YAP upregulated glucose transporter 1 (GLUT1), and inhibition of GLUT1 blocked YAP-induced hypertrophy but worsened heart function. These findings suggest that YAP is a regulator of metabolic reprogramming in the heart during compensatory hypertrophy. This insight may help in the development of future therapies for heart failure.

## A metabolic switch during cardiac remodeling

Cardiomyocyte hypertrophy is a well-described histological feature of cardiac remodeling in response to physiological or pathological stresses and involves complex molecular and cellular reprogramming. Over the past decade, substantial progress has been made in uncovering a fetal-like metabolic switch in hearts during hypertrophic remodeling, whereby fuel utilization shifts from fatty acid oxidation to glycolysis (1). However, many key questions regarding the pathophysiological role of such a fuel switch and the responsible regulatory network remain unanswered. Indeed, compensatory hypertrophy is thought to protect against pathological deterioration and avert heart failure, however, a metabolism-targeted therapy that counters pathological cardiac remodeling and heart failure has yet to be developed.

In this issue of the *JCI*, Kashihara et al. (2) report that Yes-associated protein 1 (YAP), a well-characterized transcription factor central to cellular growth and oncogenesis, also potently regulates glycolysis in cardiomyocytes. More important, the authors demonstrated that YAP-mediated glycolysis induction was necessary for cardiac hypertrophy and to preserve cardiac function under pressure overload, thus establishing a critical role for the YAP/glycolysis axis in the development of compensatory hypertrophy. YAP is a highly conserved nuclear effector for the Hippo pathway. It was first implicated in cell proliferation and organ size regulation, but it also regulates metabolism (3, 4). In cancer cells, YAP activity is essential for the induction of most of the genes involved in glycolytic pathways, which are thought to be necessary to meet the heightened demand for de novo synthesis of fatty acids, cholesterol, and amino acids (5). These aerobic and anaplerotic activities may contribute to the so-called Warburg effect observed in cancer tissues, in which increased glycolytic flux is a necessary hallmark of metabolic reprogramming for tumor growth and is exploited by clinicians to diagnose and treat cancer (6). Recently, a similar scheme of glycolysis-driven growth in cardiomyocytes has also been recognized in the process of hypertrophy, but the key upstream regulators and downstream pathophysiological impact remain to be fully elucidated (7).

## YAP/glycolysis axis

**Figure 1:**
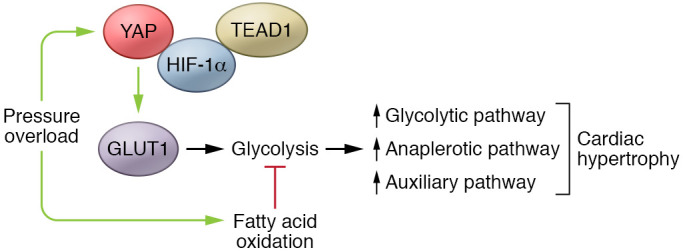
Model for YAP-mediated compensatory hypertrophy via metabolic switching. Cardiac stress, induced by pressure overload, activates YAP to bind the GLUT1 proximal promoter with TEAD1 and HIF-1α. In cardiomyocytes, GLUT1 gene transcription induces glycolysis, shifting cardiomyocytes from fatty acid metabolism. This switch to glycolysis associates with expression changes in the glycolytic, anaplerotic, and auxiliary pathways, resulting in cardiac hypertrophy (2).

Sadoshima’s laboratory previously reported that pressure overload induces YAP expression. Conversely, cardiomyocyte-specific genetic attenuation of YAP expression inhibits cardiac hypertrophy but promotes heart failure, thus implicating a role for YAP in compensatory hypertrophy (8). In the current study, Kashihara et al. further explored the metabolic reprogramming involved in the cardiac hypertrophy setting (2). The authors observed elevated glycolytic activity following pressure overload in WT mouse hearts. Importantly, this change was attenuated in the cardiomyocyte-specific YAP-deficient heart. Through targeted gene expression and metabolomics analyses, the authors found that pressure overload in cardiomyocytes triggered dynamic changes in YAP activity. YAP activity correlated with and was necessary for the altered expression of many genes in glycolytic and auxiliary pathways, as well as the changes in intra-cardiac levels of their corresponding metabolites. The in vivo observation was replicated in primary myocytes in culture, supporting the idea of a mechanism for cell-autonomous regulation in cardiomyocytes. Consistent with a role for YAP in glycolytic regulation, ^13^C tracing data showed increased production of glycolytic and anaplerotic metabolites in YAP-activated myocytes. Furthermore, the authors found that silencing of specific glycolytic and related genes had a different impact on YAP-induced hypertrophy in cultured myocytes. In particular, the anaplerotic genes associated with phosphoenopyruvate, malate, and serine production were shown to be essential for YAP-induced hypertrophy. The researchers validated the finding in vivo by inhibiting glucose transporter 1 (GLUT1) in the intact mouse heart, which blocked YAP-induced hypertrophy. Conversely, GLUT1 expression restored cardiac hypertrophy and improved function following pressure overload in the YAP-deficient mouse heart. Finally, reporter- and ChIP-based analysis revealed that YAP directly bound to the *Glut1* proximal promoter in cooperation with its known cofactors TEAD1 and HIF-1α to regulate *Glut1* gene transcription. These lines of evidence support the conclusion that YAP critically regulates glycolysis induction in cardiomyocytes and is necessary for compensatory hypertrophy (ref. 2 and Figure 1). The demonstration that YAP has a role in the glycolytic switch during cardiac hypertrophy advances our current understanding of metabolic regulation in pathological cardiac remodeling. While conceptually consistent with earlier studies of tumorigenesis, the study by Kashihara et al. offers the first comprehensive in vivo and in vitro evidence that YAP may also serve as a pivotal link to coordinate a metabolic switch in the heart under growth stimulation (2). Although the current study focused exclusively on pressure overload–induced hypertrophy in adult myocytes, the importance of the Hippo/YAP pathway in cardiomyocyte regeneration makes plausible the hypothesis that YAP-mediated glycolysis regulation may also have a critical role in cardiac development, regeneration, and repair.

## Clinical implications

Earlier studies found distinct characteristics in hypertrophic hearts induced under physiological and pathological conditions, such as exercise or pressure overload. These observations led to the current concept that myocyte hypertrophy can be adaptive and beneficial or pathogenic and deleterious to the heart, depending on the nature of the triggering stresses. This study from Sadoshima’s laboratory suggests that YAP-glycolysis regulation is essential to cardiomyocyte hypertrophy but that blocking it leads to a detrimental outcome for cardiac function (2). Although the data are consistent with the conclusion that YAP-mediated glycolysis induction is a cardioprotective process necessary to fuel compensatory hypertrophy, other studies from Rong Tian’s laboratory would argue that glycolysis induction may also fuel pathological hypertrophy and lead to a detrimental outcome (9). By augmenting or preserving fatty acid oxidation in the mouse heart, the Tian laboratory found that suppressing glycolysis induction actually contributes to an attenuated state of cardiac hypertrophy and improves cardiac function under pathological stress (9). It can be argued that both YAP expression and fatty acid oxidation induction can have multilayered effects beyond glycolysis regulation. The multifunctional role of YAP may be particularly relevant for this study, since YAP is a potent transcription regulator for many target genes. Further investigation would be needed to clarify whether glycolysis induction per se has different roles in physiological versus pathological hypertrophy, and whether glycolysis-fueled cardiomyocyte growth is truly compensatory or detrimental. Obviously, addressing this issue by selective targeting or manipulation of this important metabolic reprogramming to achieve clinical benefits is critical for future clinical translation.

Beyond myocytes, it is also important to recognize that YAP-mediated regulation may differ between cardiomyocytes and other cell types. Manvendra K. Singh’s laboratory recently reported that YAP regulates macrophage activation and phenotype switching in injured myocardium and contributes to pathological remodeling and dysfunction (10). They also demonstrated that YAP is essential for cardiac fibroblast activation and that fibroblast-specific inhibition of YAP attenuates cardiac hypertrophy and pathological remodeling (11). One interesting question is whether YAP-mediated glycolysis regulation is a shared mechanism involved in different cell populations in heart during cardiac remodeling. These observations suggest that YAP-mediated glycolysis in other cell types may influence the therapeutic potential for targeting YAP activity or the glycolysis pathway in efforts to treat heart failure. Future progress for this strategy may very well depend on cell-type specificity.

As revealed by Kashihara and colleagues (2), who performed gene-targeted manipulation in cultured myocytes, YAP-mediated regulation of glycolysis and auxiliary pathways is highly heterogenous. Kashihara et al. also showed that the impact of this YAP activity on cardiomyocyte metabolic flux and the role of YAP in cardiac hypertrophy appear to be quite complex. Many unknowns remain, including the cellular and molecular mechanisms involved in the direct and indirect impact of metabolic reprogramming on cardiac hypertrophy and dysfunction; the potential role of altered metabolites as signaling molecules in addition to substrates for cellular growth; the differences in metabolic reprogramming under physiological versus pathological hypertrophy; and a proof-of-concept demonstration of YAP-targeted therapy in heart failure. Indeed, the current report offers an interesting glimpse into the complex and interdependent network of metabolic reprogramming and cell remodeling in the heart. Much more information is needed to define the role of YAP and other players in metabolic regulation during the onset of cardiac hypertrophy, remodeling, and dysfunction. It is anticipated that recent advancements in omics technologies and system-level approaches, particularly concerning metabolic profiling and regulation, may hold exciting promise (12) to reveal more switches and links to connect metabolic reprogramming with cardiac remodeling in the heart and to uncover more targets for diagnosis and therapeutic intervention.

## Version 1 03/15/2022

Electronic publication
